# Neuroscience Outside the Box: From the Laboratory to Discussing Drug Abuse at Schools

**DOI:** 10.3389/fnhum.2022.782205

**Published:** 2022-05-12

**Authors:** Thereza Cristina Machado do Vale, Luana da Silva Chagas, Helena de Souza Pereira, Elizabeth Giestal-de-Araujo, Analía Arévalo, Priscilla Oliveira-Silva Bomfim

**Affiliations:** ^1^NuPEDEN, Nucleus for Research, Education, Dissemination and Neurosciences Popularization, Department of Neurobiology and Program of Neurosciences, Institute of Biology, Federal Fluminense University, Niterói, Brazil; ^2^Department of Neurobiology and Program of Neurosciences, Institute of Biology, Federal Fluminense University, Niterói, Brazil; ^3^Department of Molecular and Cell Biology, Institute of Biology, Federal Fluminense University, Niterói, Brazil; ^4^Department of Experimental Surgery, University of São Paulo Medical School, São Paulo, Brazil

**Keywords:** neuroscience, education, public policies, science outreach, drug education

## Abstract

One of the effects of the current COVID-19 pandemic is that low-income countries were pushed further into extreme poverty, exacerbating social inequalities and increasing susceptibility to drug use/abuse in people of all ages. The risks of drug abuse may not be fully understood by all members of society, partly because of the taboo nature of the subject, and partly because of the considerable gap between scientific production/understanding and communication of such knowledge to the public at large. Drug use is a major challenge to social development and a leading cause of school dropout rates worldwide. Some public policies adopted in several countries in recent decades failed to prevent drug use, especially because they focused on imposing combative or coercive measures, investing little or nothing in education and prevention. Here we highlight the role of neuroscience education as a valid approach in drug use education and prevention. We propose building a bridge between schools and scientists by promoting information, student engagement and honest dialogue, and show evidence that public policy regulators should be persuaded to support such science-based education programs in their efforts to effect important positive changes in society.

## Introduction

The abuse of substances, illicit or not, is a worldwide health problem that deserves immediate attention. According to the United Nations Office on Drugs and Crime, millions of drug users worldwide also have depression, anxiety or suicidal intentions (UNODC, 2021). Secondary consequences of drug abuse include a heightened risk of contracting hepatitis B/C and HIV and death or injuries from vehicle accidents (Olfson et al., 2018; Glei and Preston, 2020). The combined costs of these possible consequences most likely surpass the cost of most government prevention programs. In low- and middle-income countries, huge social inequalities worsen the issue and highlight the inefficiency of public policies that focus more on fighting rather than preventing drug abuse (UNODC, 2021).

Here we address neuroscience’s potential to contribute to this discussion (World Health Organization [WHO], 2004), especially in schools, a privileged environment for knowledge dissemination (Faggiano et al., 2005). Activities and programs that engage scientists, students, families, school teachers/staff, physicians and therapists engaged in rehabilitation, and members of the community or neighborhood are much more likely to have positive results than actions that aim to instill fear (i.e., fighting rather than preventing). We emphasize the importance of making the information engaging and accessible to the target audience by considering their prior knowledge and cultural/socioeconomic level, as well as creating public policies that promote and support such actions.

## The Brain Under (Drug) Pressure

The use of substances such as alcohol, marijuana, cocaine and opioids, among others, usually starts during adolescence, a period of rapid brain circuit maturation that is highly influenced by genetic and environmental factors (Fuhrmann et al., 2015; LeNoue and Riggs, 2016; UNODC, 2021). The need for independence, identity formation and peer acceptance (Bauman and Phongsavan, 1999; UNODC, 2021) make adolescence a critical period of physiological and social development characterized by an increase in risk-taking behaviors driven by the pursuit of quick rewards (Botvin and Botvin, 1992; Willoughby et al., 2013; UNODC, 2021). These behaviors are associated with an immature prefrontal cortex, an area of the brain responsible for evaluation and planning, decision making and impulse control. Since this region remains highly sensitive to external influences until around 20 years of age, drug use during adolescence can lead to significant functional and structural brain changes. These changes, in turn, exacerbate the natural “imbalance” among the regulatory frontal circuits (whose maturation is delayed relative to the cortico-limbic circuits), potentially leading to long-term problems in the areas of emotional control and reward feedback (Gogtay et al., 2004; Arain et al., 2013; Ernst, 2014), as well as an increased risk of developing drug addiction. Furthermore, adolescents seem unconcerned with the possible consequences of using psychoactive substances and a tendency to believe they are in control and could discontinue use at any point if they wish (Bauman and Phongsavan, 1999). Importantly, child drug use is associated with the later use of potentially more harmful drugs, such as heroin and cocaine (Fletcher et al., 2008).

Studies show that an individual’s family may be the root of substance use/abuse, since children and adolescents often look up to their parents and caregivers (Flay et al., 1994; Biederman et al., 2000). Factors that increase children’s risk of becoming smokers include free access to cigarettes (Kim et al., 2009), having parents who ask them to bring them cigarettes (Hill et al., 2005) or are heavy smokers (Hill et al., 2005), and being exposed to smoking by others over a period of years (Mays et al., 2014). Furthermore, observing relatives’ social behavior may send children the message that enjoyment is directly related to alcohol consumption (Ryan et al., 2010; Smit et al., 2018).

Alcohol abuse in young people is highly influenced by underlying levels of stress, anxiety, and depression, and can increase the chances of dropping out of school (Henkel, 2011; World Health Organization [WHO], 2014; Tice et al., 2017; Valkov, 2018). Moreover, legal and illegal drug consumption profoundly impacts young people’s mental health, a focus of great concern among health professionals, families, and public institutions (Galvão et al., 2017). These issues have become particularly important in recent months, since the beginning of the COVID-19 pandemic, which among other social disruptions, led to school closures (Cowie and Myers, 2020; Chaffee et al., 2021).

In many countries around the world, including Brazil, depression is higher among individuals who abuse alcohol, one of the first drugs that adolescents have contact with. Depression, in turn, can lead to suicidal ideation (Kim, 2017; Rehan et al., 2017; McHugh and Weiss, 2019). Data have shown that 5% of Brazilians have attempted suicide at least once and that 24% of those cases are associated with alcohol consumption. Importantly, suicide is the 3rd cause of death globally and Brazil is among the top 10 countries with the highest suicide rates (Laranjeira et al., 2012; Barbosa and Teixeira, 2021).

## The Impact of Drug Abuse on Education

In Brazil, government data suggest that drug use is prevalent among both private and public school students, with public school students usually consuming heavier, lower-cost drugs. Interestingly, students in both groups lack an understanding of substance composition, as well as the risks and consequences of drug use (Laranjeira et al., 2012).

In terms of neurological effects, evidence indicates that young people who binge drink or consume heavy amounts of alcohol show reduced gray matter in frontolateral and temporal cortices, as well as reduced white matter development in the corpus callosum and pons, which may increase the risk of developing alcohol-related disorders, as mentioned above (Squeglia et al., 2015; Cservenka and Brumback, 2017). Drug use during this period of life can also disrupt motivation, memory, and learning (Fowler et al., 2007), all functions essential during the educational process.

Excessive alcohol consumption also affects brain regions involved in visual working memory, which can have a significant negative effect on learning (Squeglia et al., 2012). Interestingly, adolescents undergoing rehabilitation for alcohol dependence show worse performance on verbal and non-verbal memory tasks relative to controls, as well as reduced hippocampal volume (Brown et al., 2000; Tapert and Schweinsburg, 2005). These structural and functional changes are bound to have long-lasting effects.

Tobacco use has been shown to increase the risk of addiction to other substances. Human and animal studies of tobacco use show impairments in learning capacity, memory, attentional control, mood, impulse control, and behavioral problems, even when consumed in small doses (Abreu-Villaça et al., 2003; Counotte et al., 2009; Gould and Leach, 2014; US Department of Health and Human Services, 2016; Valentine and Sofuoglu, 2018; Zarrindast and Khakpai, 2019; Leslie, 2020). While tobacco use among young people has been a concern for several decades, the relatively recent “new wave” of flavored e-cigarettes has worsened the problem, as these are particularly popular among teenagers (Brown et al., 2000). While flavored e-cigarettes are mostly marketed to people wanting to quit, users are often young people who never smoked before and who mistakenly believe that e-cigarettes are less harmful than conventional cigarettes (Flay et al., 1994; Biederman et al., 2000; Kim et al., 2009). Besides nicotine, e-cigarettes contain substances- some with carcinogenic, pro-inflammatory and immunosuppressive potential- whose short and long-term effects are still unknown (Hill et al., 2005; Mays et al., 2014).

Alcohol and nicotine are thought to be the gateway for illegal drugs like Cannabis (Secades-Villa et al., 2015), which also impairs learning, memory and attention. Most of these effects are dose-dependent, with considerable interindividual variation (D’Souza et al., 2008; Ramaekers et al., 2009; Theunissen et al., 2012; Petker et al., 2019). Chronic marijuana use in adolescents can lead to irreversible IQ loss, even when use is interrupted in adulthood (Meier et al., 2012). Also, Owens et al. (2019) reported that a positive urine screen for THC was associated with lower performance on working memory tasks, as well as reduced fMRI activity in the prefrontal cortex, posterior parietal cortex, supplementary motor area, and insula, even when there was no previous history of cannabis use. Furthermore, since learning consolidation is aided by positive emotions (Tyng et al., 2017) and marijuana is involved in the processing of negative emotions (Bossong et al., 2013), its use may play an additional negative role in learning.

Finally, as mentioned above, one major consequence of substance abuse in school-aged children (besides the cited neurophysiological, social and emotional issues) is an increased school dropout rate (Tice et al., 2017; Valkov, 2018), a critical problem in Brazilian education that is also fueled by inefficient public policies, family disruption, and learning difficulties, among other factors (Vasters and Pillon, 2011; Cardoso and Malbergier, 2014; Bittencourt et al., 2015; de Silva Filho and Araújo, 2017).

## Neuroscience as an Ally to Education: A Tool to Prevent Use/Abuse

Neuroscience knowledge can be used to help kids and teens foster healthy cognitive and emotional skills that will help them make choices regarding drug use and other behaviors. However, it is often challenging to present this knowledge in a way that is accessible and easy to relate to, and one must always consider individuals’ prior knowledge and experience (Bruer, 1997; World Health Organization [WHO], 2004; Sigman et al., 2014; Horvath and Donoghue, 2016). The choice of language is critical, as oversimplifications could generate more neuromyths (Howard-Jones and Fenton, 2012). Furthermore, one must also consider the audience’s expectations and concerns, their religious and ethical values, and their socio-economic status.

**FIGURE 1:**
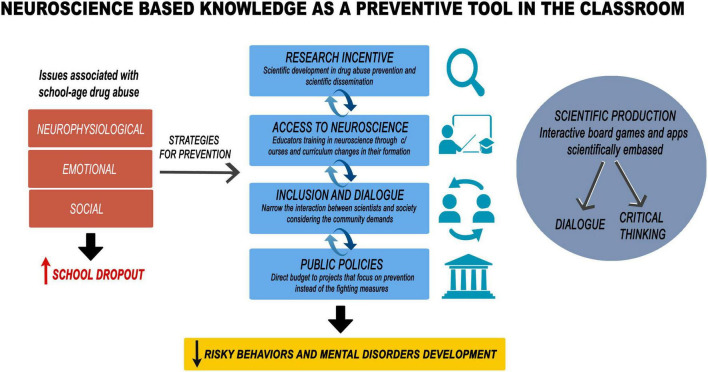
This figure illustrates our view of what a successful educational drug abuse prevention program should include. On the left side, we list the main issues associated with school-age drug abuse, which include neurophysiological, emotional, and social factors that are known to increase rates of school dropout. On the right side of the figure are efforts of scientific production, including interactive games and courses, which generate dialogue and critical thinking and, in turn, promote research incentives, access to neuroscience, inclusion and dialogue, and public policies (middle panel), all of which can help reduce risky behaviors and mental disorders.

Since most research is published in English, an added challenge for scientists and other professionals in non-English speaking countries is translating scientific findings (as well as adapting them) for a non-English speaking audience (Márquez and Porras, 2020; Roche et al., 2020). A major aim of scientific communication is to reach people who do not work in the areas of science or health and provide them with the opportunity and tools to understand and discuss issues that are of interest to society, such as drug abuse (Fischhoff, 2013). Figure 1 represents a model of what this process should include, from scientific information adapted by scientists for proper communication to the elaboration/modification of targeted public policies.

In line with this proposal and following UNESCO’S recommendation for educators to apply interactive teaching methods (UNESCO et al., 2017), our group previously developed a neuroscience-based board game called “Crash: find the exit” to promote information, engagement and dialogue at schools about substance abuse and its effects on the central nervous system (Da Silva Chagas et al., 2020). In this collaborative game developed for middle and high school students (ages 12 and older), we present information about 22 neuroactive drugs (e.g., depressants, hallucinogens, stimulants, anabolic steroids, and prescription drugs) as well as brain anatomy, cell types/structures, neurotransmitter actions, the impact of drug abuse on nervous system physiology, and the mechanisms underlying overdose (including effects on other systems, such as cardiovascular and renal). We also added discussion points about depictions of drug use in series or films, making it a particularly interesting experience for young audiences (Da Silva Chagas et al., 2020). Other initiatives for teaching young people about drug abuse have also employed games and even interactive platforms (Miller et al., 2006; Cheng et al., 2011; Klisch et al., 2013; Epstein et al., 2016; Kapitány-Fövény et al., 2018; Stapinski et al., 2018). Such projects have shown positive results in reducing first use, preventing transition to addiction, and overall prevention (Faggiano et al., 2014).

Importantly, interventions are most effective if they are tailored to the group’s particular age and risk level (Gilligan et al., 2019). According to MacArthur and colleagues, school programs target normative beliefs (i.e., beliefs held by peers or other important people that influence kids’ behaviors), establish a bond with the school, and train risky behavior avoidance. However, this approach does not establish a dialogue or promote adolescents’ critical thinking or their ability to make choices. Instead, it aims to impose socially desirable behaviors while avoiding those endorsed by peers (Silva, 2016; MacArthur et al., 2018).

According to Faggiano and colleagues, there are three reasons why schools are the right environment to implement drug-prevention programs: (1) four out of five tobacco smokers start before adulthood, thus substance use prevention should focus on school-age children and adolescents before their beliefs and expectations about substance use are established; (2) schools offer the most systematic and efficient way to reach significant numbers of young people each year, and (3) in most countries, schools can adopt and enforce a wide range of educational policies (Faggiano et al., 2005). While other types of interventions have been proposed, including those targeting individuals and families (Carney et al., 2016; MacArthur et al., 2018), evidence indicates that school-based interventions are more effective (Faggiano et al., 2005, 2014; Fletcher et al., 2008; Carney et al., 2016; Lichtenberg et al., 2020).

As discussed by Sigman et al. (2014), among others, social development requires us to build a bridge between neuroscientists and school educators. Scientists develop tools, technologies and educational approaches that engage young people and promote experiences that consolidate learning. School educators, in turn, can use their expertise in dealing with different age groups to disseminate the information in a palatable and efficient manner (Howard-Jones and Fenton, 2012). We advocate in favor of a two-way partnership between researchers and teachers or research centers and schools aiming to find creative alternatives to presenting important information (e.g., about drug abuse^1^). These approaches should be as fun, interactive and engaging as possible (Da Silva Chagas et al., 2020).

Importantly, one should always engage the participation of doctors, therapists and other health workers directly involved in drug abuse treatment, as these professionals can educate the public about the negative impact of drug abuse not only on physical and mental health, but also on individuals’ social well-being (e.g., work and relationships). Thus, by sharing their real-life experiences—which are usually not described in scientific articles—health professionals add a fundamental piece to the drug-abuse conversation started by scientists, family members, educational staff, and society. In essence, they can add yet another ‘human’ component to complement the statistics (Mills and Wonoprabowo, 2020).

Another important strategy is to be welcoming and engaging toward families, as several studies have reported an association between child/adolescent substance use/abuse and their parents’ own history of substance and/or psychiatric disorders, two variables included in the list of family-based Adverse Childhood Experiences (ACEs) (Forster et al., 2018; Shek et al., 2020).

## Public Policies: Combative vs. Preventive Strategies

In 2018, an estimated 269 million people worldwide had used drugs at least once (UNODC, 2021). In Brazil, among 50,890 interviewed public and private elementary and high school students from all 27 Brazilian capitals, this number was 13,000, or 25.5% (see text footnote 1). These high numbers may be attributed to inefficient policies implemented in Brazil, which, as mentioned before, favor a combative and prohibitive approach, rather than one centered on education and prevention (VI LENADE, 2010). In 2017, one billion reais (approximately 186 million US dollars as of August 2021) were invested in anti-drug laws by the state of Rio de Janeiro. This amount would be enough to fund the education of 252,000 high school students or 32,000 public university students for an entire year or build 121 schools for more than 77 thousand new high school students (Lemgruber et al., 2021). These data suggest that money allocated to “fight drugs” (i.e., costs associated with public safety, public ministry and defense, the rehabilitation of juvenile offenders, and the prison system) might be better used in preventive programs. Scientists and educators should join forces to develop programs that follow this line of thinking and that meet the needs of communities from different socioeconomic levels and cultures in different parts of the world.

However, not all prevention-based programs are successful. In the US, two well-known campaigns–“Just Say No” and its byproduct DARE (Drug Abuse Resistance Education)–were developed in the early 80s and were implemented for many decades. Despite extraordinary government investments and insistence on these programs, it is generally known that they failed at what they proposed to do (Pan and Bai, 2009; Lilienfeld and Arkowitz, 2014). While the approach was preventive and not combative, the ‘‘Just Say No’’ campaign did not provide any useful information or tools for young people to make the right decisions autonomously. While the DARE campaign^2,3^ did involve some drug-related education, its approach was never inclusive or interactive. The same failure has been observed with a Brazilian adaptation of these programs, a project called PROERD (Educational Program for Drug Resistance and Violence) (Sanchez et al., 2021), which has traditionally followed a more combative approach. In sum, none of these programs invite young people to think, judge, evaluate or make their own decisions.

On the other hand, positive results have been observed with the application of some non-normative programs in Europe. A study conducted simultaneously in Austria, Belgium, Germany, Greece, Italy, Spain, and Sweden aimed to prevent/avoid the use (experimental and regular) of alcohol, tobacco, and illicit drugs among students 12–14. In this 1 h/week in-school intervention over 12 weeks (with an 18-month follow-up), students learn about different substances and search for the associated toxic properties and physiological alterations. This study resulted in a reduction of about 38% for alcohol use and 26% for cannabis use (Faggiano et al., 2010). Moreover, other reviews covering 29 and 51 US-based studies, respectively (Faggiano et al., 2005, 2014) indicate that school-based programs produce a Number Needed to Treat (NNT) ratio of 33 for marijuana use (i.e., one out of every 33 students will be positively influenced by the intervention and will choose not to smoke marijuana), which is considered successful compared to similar studies.

More recently, programs like those cited above have been gradually implemented in school curricula in some countries. In the US, the “Safety first Program” includes 15 45–50 min classes containing teacher-guided interactive activities providing information regarding substances, their effects and drugs policies. This program is part of Drug Policy Alliance (see text footnote 2), a US-based policy that trains high school teachers to converse openly with their students to help them make better drug-related choices. In Australia (see text footnote 2) a similar initiative is implemented from the beginning of the educational process as part of the “health and physical education” curriculum. The goal is to promote a discussion about drugs and their impact on different levels, including personal, familial and community. This Australian Program revisits the school curriculum early on (foundation to year 10), providing young people with honest and scientifically accurate information that is age-appropriate and thus enables them to evaluate information critically and consciously. Following educational public policies, successful programs cover topics such as HIV transmissibility (needle sharing), teenage pregnancy (considering the impact of drugs in fetal development), chemical dependence, mental health disorders, and the consequences of car accidents (e.g., resulting from drunkenness), all potential consequences of drug use/abuse (UNODC, 2021).

## Conclusion

While, there is some disagreement on the potential contribution of using neuroscience-based knowledge in the classroom (and how such contributions should be implemented) (Bruer, 1997; Sigman et al., 2014; Horvath and Donoghue, 2016), we strongly believe there is an effective way of using scientific knowledge in favor of social development, as has been shown with several successful projects cited above. However, it is important to emphasize that such efforts should engage teachers, parents and the community at large to (1) create a positive atmosphere that promotes open discussion; (2) avoid spreading erroneous or misleading information about drug abuse or other neuroscience concepts; (3) improve and stimulate neuroscience knowledge as a tool to prevent drug use/abuse among the youngest; (4) guide teachers and students on how to approach this topic creatively; (5) legitimize the school’s role in transforming society while providing knowledge to both children/adolescents and their communities; (6) stimulate the study/popularization of neuroscience in schools by leading important discussions about social development; (7) promote the dissemination of this content beyond the school environment to promote critical reflection in the community at large.

In this paper, we show how strategies that aim to fight drug use by instilling fear or teaching young people to avoid certain behaviors often meet little or no success, while those that foster inclusion and incite dialogue and understanding are much more effective. Conducting fun activities while teaching neuroscience in an audience-appropriate language can be an excellent tool in such efforts. In Brazil, efforts to establish a bridge between the (neuroscience) laboratory and schools as well as the community at large have been increasing in the last few years. For such efforts to work, scientists must find a way to work side by side with people in the schools (teachers, students, families, staff), listening to their needs and finding new ways to provide information through neuroscience-based activities. The development of socially innovative tools such as the board game developed by our group pave the way for new similar approaches. In addition, the discussion about issues like drug abuse needs to be encouraged at home as well. The first critical step is to start the dialogue by making information accessible and motivating scientists to engage society. These scientists can then join forces to develop new preventive strategies. For now, our aim is to expose these ideas and encourage scientists, educators and policymakers around the world to actively engage in such efforts and thus contribute to improving society’s knowledge and well-being.

## Data Availability Statement

The original contributions presented in the study are included in the article/supplementary material, further inquiries can be directed to the corresponding author.

## Author Contributions

TM, AA, and PO-S designed the study. TM, LS, HS, and PO-S managed literature searches and wrote the manuscript. EG-d-A, AA, and PO-S critically reviewed the manuscript. LS, HS, and PO-S designed the figure. All authors contributed to and approved the final manuscript.

## Conflict of Interest

The authors declare that the research was conducted in the absence of any commercial or financial relationships that could be construed as a potential conflict of interest.

## Publisher’s Note

All claims expressed in this article are solely those of the authors and do not necessarily represent those of their affiliated organizations, or those of the publisher, the editors and the reviewers. Any product that may be evaluated in this article, or claim that may be made by its manufacturer, is not guaranteed or endorsed by the publisher.

## Footnotes

1 According to data from the sixth national survey on psychotropic drug use among public and private elementary and high school students.

2 Drug Policy and Alliance (2021). https://drugpolicy.org/resource/safety-first-real-drug-education-teens

3 Australian curriculum review (2021). https://www.australiancurriculum.edu.au/f-10-curriculum/health-and-physical-education/structure/
